# Crosstalk of clock gene expression and autophagy in aging

**DOI:** 10.18632/aging.101018

**Published:** 2016-08-28

**Authors:** Faiza Kalfalah, Linda Janke, Alfonso Schiavi, Julia Tigges, Alexander Ix, Natascia Ventura, Fritz Boege, Hans Reinke

**Affiliations:** ^1^ University of Düsseldorf, Medical Faculty, Institute of Clinical Chemistry and Laboratory Diagnostics, 40225 Düsseldorf, Germany; ^2^ IUF-Leibniz Research Institute for Environmental Medicine, 40225 Düsseldorf, Germany

**Keywords:** aging, circadian clock, autophagy, primary human skin fibroblasts, lin-42, *C. elegans*

## Abstract

Autophagy and the circadian clock counteract tissue degeneration and support longevity in many organisms. Accumulating evidence indicates that aging compromises both the circadian clock and autophagy but the mechanisms involved are unknown. Here we show that the expression levels of transcriptional repressor components of the circadian oscillator, most prominently the human Period homologue *PER2*, are strongly reduced in primary dermal fibroblasts from aged humans, while raising the expression of *PER2* in the same cells partially restores diminished autophagy levels. The link between clock gene expression and autophagy is corroborated by the finding that the circadian clock drives cell-autonomous, rhythmic autophagy levels in immortalized murine fibroblasts, and that siRNA-mediated downregulation of *PER2* decreases autophagy levels while leaving core clock oscillations intact. Moreover, the Period homologue *lin-42* regulates autophagy and life span in the nematode *Caenorhabditis elegans*, suggesting an evolutionarily conserved role for Period proteins in autophagy control and aging. Taken together, this study identifies circadian clock proteins as set-point regulators of autophagy and puts forward a model, in which age-related changes of clock gene expression promote declining autophagy levels.

## INTRODUCTION

Several hallmarks of aging are related to autophagy, which is a mechanism protecting from chronic organ degeneration and prolonging life span by contributing to proteostasis and mitochondrial homeostasis [1]. The process of autophagy regulates many aspects of metabolic homeostasis and stress response and involves the inclusion of cellular components into vesicles called autophagosomes, which then fuse to the lysosome, the principle organelle for bulk degradation in eukaryotic cells. Autophagic flux decreases with age in many organs [2], whereas its upregulation is involved in life span extension by mitochondrial stress-adaptation and caloric restriction [3]. Genetic restoration of autophagy improves liver function in aged rodents, and pharma-cological stimulation of autophagy prolongs the life span of various model organisms [4, 5].

Another prominent mechanism that affects the mammalian aging process is the circadian clock. It confers rhythmic gene expression to a large part of the genome, which leads to overt rhythmic changes in physiology and behaviour. In the centre of the mammalian circadian clock lies a negative feedback loop of Period (*PER*) and Cryptochrome (*CRY*) gene expression, which is activated by the transcription factors Aryl hydrocarbon receptor nuclear translocator-like (*ARNTL*/*BMAL1*) and Circadian locomotor output cycles kaput (*CLOCK*). All body clocks are under the control of a light-sensitive master oscillator in the suprachiasmatic nucleus (SCN) of the hypothalamus, and additionally receive input from a variety of metabolic signals informing on physiological parameters such as energy and redox levels or body temperature [6]. Circadian clock genes regulate several pathways involved in life span modulation and organ aging. These include DNA repair and antioxidative or xenobiotic defence systems [7, 8]. Robust functionality of the molecular clockwork is linked to the cellular redox metabolism and declines with age in the master clock of the SCN [9]. Mouse models defective in specific clock genes have a shortened life span and exhibit features of accelerated organ aging [10]. In humans, disruption of circadian rhythmicity is associated with an earlier onset of metabolic and cardiovascular dysfunction. It is therefore evident that both autophagy and the circadian clock are crucial to counteract aging. However, if and how these mechanisms are connected in aged cells and if key regulators exist that coordinate the circadian clock and autophagy to promote longevity is an important unresolved question.

## RESULTS

### Decreased autophagy levels in aged primary human skin fibroblasts

**Figure 1:**
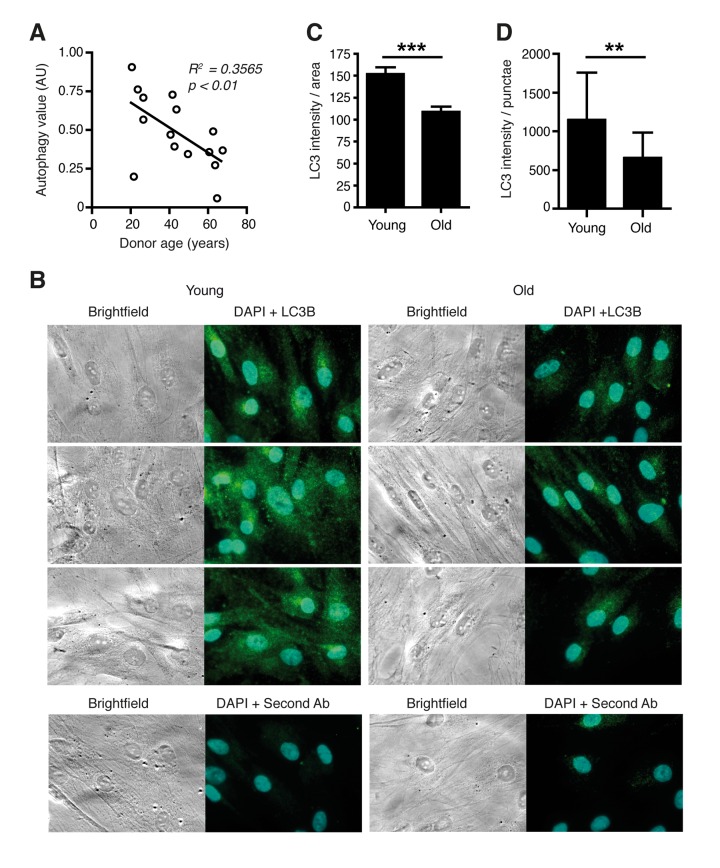
Autophagic flux is reduced in aged primary human fibroblasts (**A**) Correlation of age and LC3-II protein levels in primary dermal human fibroblasts from differently aged donors. AU: artificial units. (**B**) Immunostaining of LC3B protein in cells from young and old donors after Bafilomycin treatment. In the bottom row cells stained only with secondary antibody (Cy3) and DAPI are shown. Total signal strength (**C**) and the number of LC3 positive punctae in a defined area (**D**) were quantified.

We chose to study the link between the circadian clock, autophagy and aging in dermal fibroblasts. Skin cells are subjected to actinic stress leading to protein oxidation and mitochondrial dysfunction, which are typically cleared by autophagy [11]. Moreover, mice that are challenged by UV irradiation at different times of the day display a varied multiplicity of sunburn apoptosis, erythema, and skin cancer [7]. First, we tested the hypothesis that changes of molecular oscillator functions might entail a decline of autophagy levels during the mammalian aging process. For this purpose an established cellular ageing model was employed that consists of primary fibroblasts derived from surgical skin samples of human donors aged 20 – 67 years. Skin samples were obtained during female breast reduction surgeries ruling out tissue- or sex-specific effects in the analyses. Cells were analysed well below the replication limit to exclude possible influences of replicative senescence, which was demonstrated by comparable levels of β–galactosidase staining in cells from young and old donors with the same passage numbers (Fig. S1) and had been shown with various markers in previous studies [12, 13]. The model has been demonstrated to display several features typically associated with skin aging including altered protein secretion, altered hyaluronidase activity, altered gene expression, decreased proliferative capacity, decreased genome stability and impaired mitochondrial function [12-14]. Autophagic flux was measured by determining the levels of the lipidated form of Microtubule-associated protein 1 light chain 3 (LC3-II), a generally accepted marker for autophagosome formation [15]. LC3-II levels without treatment with the lysosome inhibitor Chloroquine were subtracted from LC3-II levels in the presence of Chloroquine to determine a value for autophagic flux [16]. Autophagic flux values correlated strongly to the age of the donors (Fig. 1A) whereby a difference between the highest value in a donor aged 21 years and the lowest value observed in a donor aged 67 years of more than 10-fold was observed, but a large scatter within biological samples of similar age indicated a marked impact of biological diversity on this parameter. Additionally, cellular LC3 levels measured by immunostaining (Fig. 1B-C) and, more importantly, the number of LC3B positive punctae revealing LC3B molecules engaged in the autophagy process (Fig. 1B and D) were reduced in cells from aged donors after lysosome inhibitor treatment. Thus, in human dermal fibroblasts, autophagy appears to decline with age in a similar fashion as in other human organs and cell types [2, 17, 18].

### Clock gene expression, autophagy levels, and donor age are correlated in primary human skin fibroblasts

**Figure 2:**
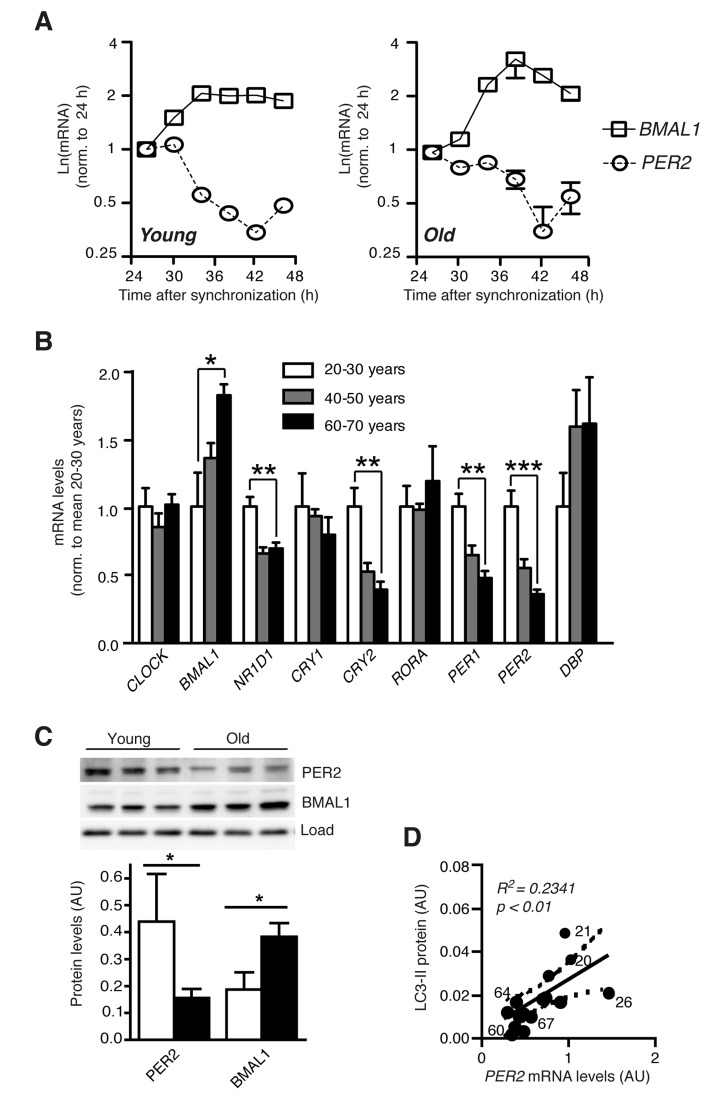
Clock gene expression is deregulated in aged primary human fibroblasts (**A**) Circadian expression of *Bmal1* mRNA (squares) and *Per2* mRNA (circles) in primary human dermal fibroblasts from one young (age 21) and one old (age 67) donor of the cohort. (**B**) mRNA levels of core clock genes in cell lines from differently aged donors. Data obtained for age groups 20-30 years (white), 40-50 years (grey) and 60-70 years (black) are shown as mean ± SEM of five individual donors per age group. Data are normalised to the mean of age group 20-30 years. Asterisks indicate statistically significant differences between age groups 20-30 years and 60-70 years (unpaired t-test, two-tailed). (**C**) PER2 and BMAL1 protein levels in age groups 20-30 years (white) and 60-70 years (black). (**D**) Correlation of *PER2* mRNA expression with LC3-II protein levels in the same cell lines. Solid and dashed lines indicate the linear regression curve and the 95% confidence band. Numbers next to data points show donor ages. Data are shown as mean values ± SEM, n=4.

We then asked whether reduced autophagic flux in cells from old donors might be linked to a defective circadian oscillator. However, in both young and aged cells the circadian clock showed highly rhythmic behaviour as determined by mRNA expression of the endogenous *BMAL1* and *PER2* genes (Fig. 2A) and luciferase activity driven by the circadian *BMAL1* promoter after lentivirus transduction (Fig. S2) [19, 20], excluding a total loss of circadian regulation as a major detrimental factor in aged fibroblasts. Next, we addressed the possibility that expression changes of individual clock genes rather than breakdown of the oscillator as a whole might play a role in age-related changes of autophagy regulation. The determination of core clock gene expression levels in the same set of cells revealed a significant age-related deregulation of a subset of genes. In particular several genes of the negative and interconnecting limbs of the clock (*PER1*, *PER2*, *CRY2*, *NR1D1*) exhibited age-related down regulation at the mRNA level, while *BMAL1*, a member of the positive limb, was the only gene exhibiting an increase in expression in aged fibroblasts (Fig. 2B). Analogous to the changed mRNA levels, BMAL1 protein expression was increased and PER2 protein expression was decreased in primary fibroblasts from old donors (Fig. 2C). Moreover, a highly age-dependent linear relationship existed between autophagic flux as determined by LC3-II accumulation after lysosome inhibition and the mRNA expression levels of *PER2* (Fig. 2D) and *BMAL1* (Fig. S3A). In addition, mRNA expression of *CRY2*, *PER1* and *NR1D1* was similarly correlated to donor age and LC3-II levels (Fig. S3B-D), while no such correlation could be detected for the mRNA abundance of *CRY1*, *RORA*, *CLOCK* or *DBP* (Fig. S3E-H). Therefore the deregulation of individual clock genes rather than dysfunction of the molecular oscillator as a whole might contribute to the decline of autophagy in aged human fibroblasts.

**Figure 3:**
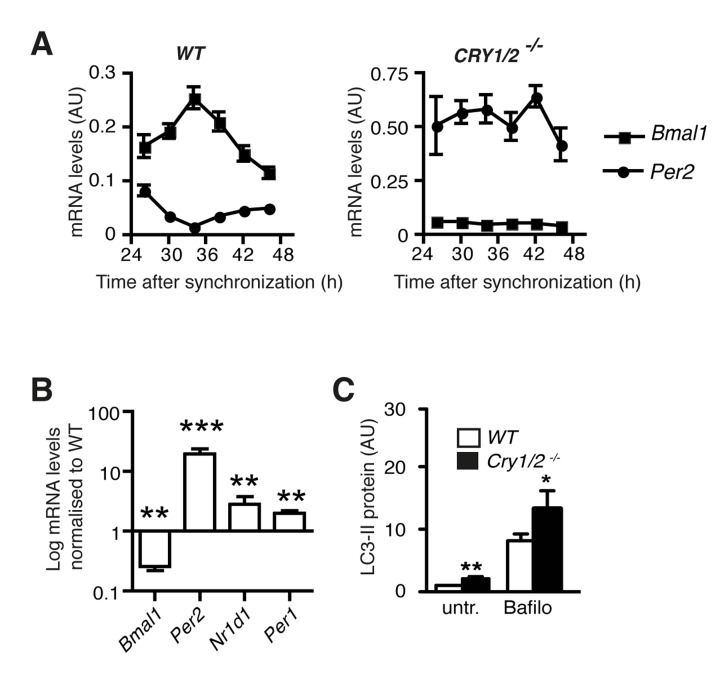
Autophagy in Cryptochrome-deficient MEFs (**A**) Circadian accumulation of *Bmal1* mRNA (squares) and *Per2* mRNA (circles) in synchronised wild type and *Cry1/2*^−/−^ MEFs. (**B**) *Bmal1*, *Per2, Nr1d1 and Per1* mRNA levels in *Cry1/2*^−/−^ MEFs normalised to the corresponding values in wild type MEFs. Data are shown as mean values ± SEM, n=8. (**C**) Autophagic flux in wild type MEFs (white) and *Cry1/2−/−* MEFs (black) determined by quantification of LC3-II protein levels in the absence or presence of bafilomycin (Bafilo).

In order to corroborate the finding that autophagic flux is correlated to the expression levels of individual core clock components we analysed autophagy in mouse cells deficient in both Cryptochromes *Cry1* and *Cry2*. As expected, lack of *Cry1* and *Cry2* leads to the loss of rhythmic gene expression in synchronized fibroblasts (Fig. 3A) [21], but more importantly, it also results in increased levels of *Per1*, *Per2* and *Nr1d1* mRNA and decreased expression of *Bmal1* mRNA (Fig. 3B).

Therefore these cells present the opposite of aged human fibroblasts regarding the regulation of this subset of clock genes. Autophagic flux was significantly increased in *Cry1*/*Cry2*-deficient cells (Fig. 3C) and thus also in this respect presented the opposite of aged human fibroblasts, which showed a down-regulation of autophagic flux (Fig. 1). In summary, clock gene expression is correlated identically to autophagic flux in two highly different mammalian fibroblast cell lines.

### The circadian clock drives cell-autonomous oscillations of autophagy levels in mammalian fibroblasts

**Figure 4:**
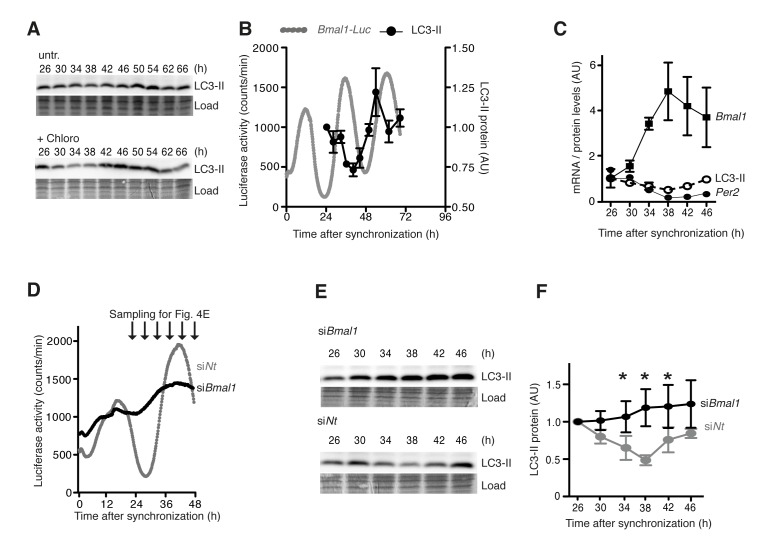
Cell-autonomous regulation of autophagy in mouse fibroblasts (**A**) Western blots of LC3-II without (upper panel) or with (lower panel) the lysosome inhibitor Chloroquine in synchronised NIH 3T3 *Bmal1*-*Luc* fibroblasts. The time after Dexamethasone treatment is indicated on top. (**B**) Quantification of LC3-II protein levels (black) and *Bmal1-Luc* reporter gene activity (grey). (**C**) Levels of endogenous *Bmal1* mRNA (squares), *Per2* mRNA (closed circles) and LC3-II protein (open circles). Data are normalised to the respective mean value at 26 h. (**D**) Circadian luciferase activity in NIH 3T3 *Bmal1-Luc* fibroblasts after transfection with *Bmal1*-specific siRNA (black) or an equivalent dose of Non-target (Nt) siRNA (grey). (**E**) Expression of LC3-II protein at time points indicated in (**D**). (**F**) Quantification of LC3-II protein levels. Data are normalised to the mean value at 26 h. Asterisks designate statistically significant differences between siBmal1 and siNt (unpaired t-test, two-tailed). All data are shown as mean values ± SEM, n=4.

These findings implied that the circadian clock might directly control autophagy levels in mammalian fibroblasts, and that autophagy levels might depend on clock gene expression levels. Circadian autophagy levels were analysed in a designated model system for circadian rhythmicity in cultured cells, namely murine NIH 3T3 cells, in which the cellular clock had been synchronized by dexamethasone treatment [22]. Autophagy was measured by quantification of LC3-II. Circadian oscillations were measured in the same cells as luciferase (*Luc*) activity driven by the circadian *Bmal1* promoter from a stably integrated transgene [23]. Under these conditions rhythmic changes in LC3-II levels with a period length of around 24 hours were observed (Fig. 4A-B). A minimum of LC3-II levels at ~38 h was followed by an increase and a maximum at ~50 h. A long exposure of the LC3-II-Western blot revealed the slower migrating LC3-I band (Fig. S4A). Autophagic flux was also determined by measuring LC3-II accumulation after lysosome inhibition, which was likewise highly rhythmic in synchronized cells (Fig. 4A, lower panel). Circadian LC3-II accumulation was antiphasic to the expression of endogenous *Bmal1* mRNA and in phase with *Per2* mRNA expression (Fig. 4C). In support of this finding we observed rhythmic accumulation of the protein Unc-51 like autophagy activating kinase 1 (ULK1) (Fig. S4B), which controls a rate-limiting step in autophagosome initiation [24]. In contrast, neither the mRNA expression of *Ulk1* nor that of several other key autophagy genes such as *Map1/Lc3b* showed a circadian pattern (Fig. S4C-I). Importantly, genetic ablation of the cellular oscillator by siRNA-mediated knock down of BMAL1, which renders cells arrhythmic (Fig. 4D), abolished the circadian accumulation of LC3-II (Fig. 4E-F). From these results we concluded that the circadian clock drives cell-autonomous oscillations of autophagy levels in mammalian fibroblasts.

#### PER2 expression regulates autophagy levels

**Figure 5:**
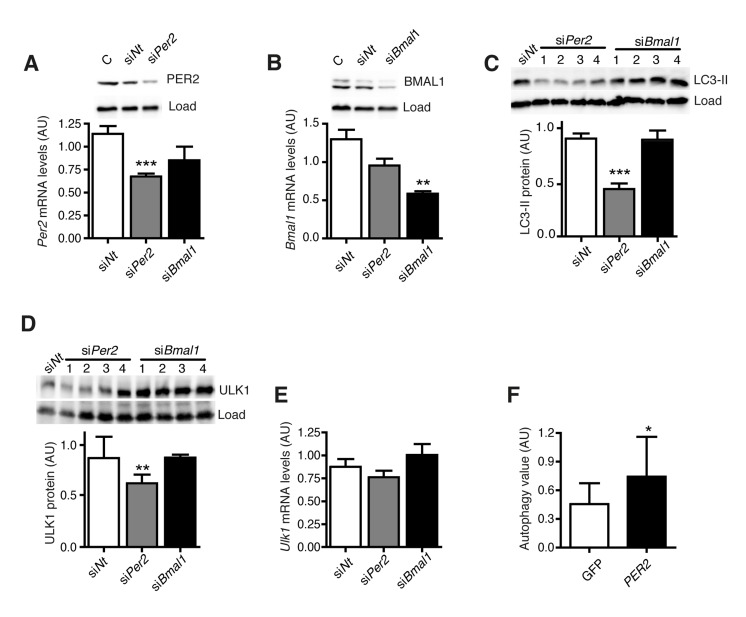
PER2 regulates autophagy (**A**) *Per2* mRNA and protein levels after transfection with *Per2*-or *Bmal1*-specific siRNA or an equivalent dose of non-target siRNA. C: untransfected control sample. (**B**) *Bmal1* mRNA and protein levels. (**C**) LC3-II protein levels. (**D**) ULK1 protein levels. (**E**) *Ulk1* mRNA levels. All data are shown as mean values ± SEM, n=4. Asterisks designate statistically significant differences of specific siRNA treatment versus control treatment with siNt (unpaired t-test, two-tailed). (**F**) Autophagy value (LC3-II in inhibitor treated cells – LC3-II in untreated cells) in two different cell lines from donors aged 60-70 years expressing only GFP (white) or PER2-GFP (black). Asterisks designate statistically significant differences of PER2-GFP versus GFP expression (unpaired t-test, two-tailed), n=3.

*PER2* and *BMAL1* were the most strongly deregulated genes in aged human fibroblasts (Fig. 2B). Therefore we asked whether these genes directly control autophagy levels. PER2 or BMAL1 were knocked down individually by siRNA-treatment in NIH 3T3 fibroblasts, and the effect on autophagy levels and autophagy gene expression was determined. Treatment with siRNA efficiently down regulated PER2 (Fig. 5A) and BMAL1 (Fig. 5B), but LC3-II levels were only reduced after knockdown of PER2 (Fig. 5C). Moreover, knockdown of PER2 altered autophagy levels while leaving core clock oscillations largely intact (Fig. S5A). This observation agrees with the findings in aged human cells (Fig. 2A-B).

Knockdown of PER2 also reduced the cellular amount of ULK1 protein (Fig. 5D) while not affecting *Ulk1* mRNA levels (Fig. 5E), which is in line with rhythmic ULK1 protein and non-rhythmic *Ulk1* mRNA levels in these cells (Fig. S4B-C). Notably, in combination these findings put forward a mechanistic basis for the correlation between deregulated clock gene expression and decreased autophagy levels in aged human fibroblasts via PER2.

We then asked whether diminished autophagy levels in dermal fibroblasts from old human donors could be rescued by restoring core clock gene expression towards the levels observed in cells from young donors. PER2-GFP was transiently overexpressed in primary dermal fibroblasts from old human donors and autophagic flux was determined. As expected, PER2-GFP displayed distinct patterns of subcellular localization reflecting various states of its regulation by nuclear import, whereas GFP alone was evenly distributed in the cells (Fig. S5B). Importantly, expression of PER2-GFP increased autophagic flux compared to cells that were transfected with a control vector expressing only GFP (Fig. 5F). This finding provides further evidence that altered core clock gene expression contributes to the age-related reduction of autophagic flux and might thereby promote the aging process of human skin fibroblasts. It also demonstrates that the dysfunctional autophagy mechanism of aged cells can be “rejuvenated” by elevating the expression of a single core clock gene.

### The *C. elegans* Period homologue Lin-42 regulates autophagy and longevity

**Figure 6:**
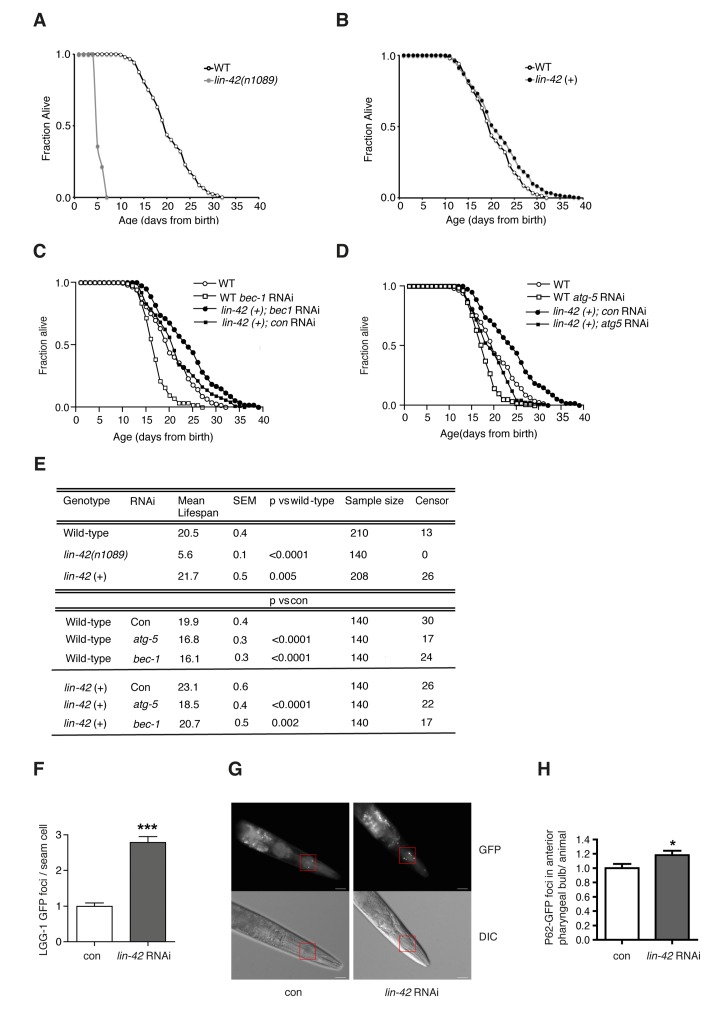
*Lin-42* regulates lifespan and autophagy in *C. elegans* Survival analyses of wild-type animals (WT) compared to *lin-42(n1089*) mutants (**A**) or to *lin-42* (+) transgenic animals (**B**). Survival analyses of wild-type animals (WT) and *lin-42* (+) transgenic animals fed bacteria transformed with empty-vector (*con*) or with vector expressing dsRNA against *bec-1* (**C**) or *atg-5* (**D**). (**E**) Table summarizing survival data analysis from (**A**-**D**). (**F**) Quantification of LGG-1/LC3::GFP positive foci in the DA2123 transgenic strain fed bacteria transformed with empty-vector (*con*) or with vector expressing dsRNA against *lin-42* (*lin-42* RNAi). Results are plotted as mean ± SEM of GFP foci (***) represent the P-value (<0.0001) calculated by performing the t-test between the 2 conditions. **(G**) P62/SQST-1::GFP translational reporter strain (HZ589) fed bacteria transformed with empty-vector (*con*) or with vector expressing dsRNA against *lin-42* (*lin-42* RNAi). Top panels: green fluorescence channel (GFP) images. Bottom panels: differential (Nomarski) interference contrast images (DIC). Red squares indicate selected areas used for fluorescence foci quantification. White bars = 20μm. (**H**) Quantification of GFP positive foci in the anterior pharyngeal bulb of HZ589 fed bacteria transformed with empty-vector (*con*) or with vector expressing dsRNA against *lin-42* (*lin-42* RNAi). Results are plotted as normalized mean ± SEM of GFP foci, relative to the control. (*) represent the P-value (<0.05) calculated by performing the t-test between the 2 conditions.

A connection between Period genes and aging exists in animals as different as mice [25, 26] and fruit flies [27], and might point towards an evolutionarily conserved role for Period genes in the age-dependent regulation of autophagy levels. We thus turned to the nematode *Caenorhabditis elegans*, a powerful genetic tractable model organism widely used for aging studies, in which autophagy is essential for development and life span extension in different genetic backgrounds [28-30]. The post-embryonic larval development of *C. elegans* is characterized by four molting cycles, which lead to a periodic renovation of animal cuticles through the epidermal blast cells, and which are regulated by hetero-chronic genes such as *lin-42*, a homolog of mammalian *PER2* [31]. The transcript levels of *lin-42* oscillate in the epidermis during post-embryonic development with peaks during the molting time, and inactivation of *lin-42* leads to arrhythmic molting and causes abnormal differentiation of epidermal stem cells [31, 32]. To reveal whether altered expression of *lin-42* also regulates *C. elegans* aging we assessed the life span of animals with mutant or with extra copies of *lin-42*. We observed that, similar to wild-type animals, *lin-42* mutants develop into fertile adults within three days after hatching, but after reaching the adult stage they quickly start dying (Fig. 6A and 6E).

Although we cannot rule out a developmental problem in facilitating the dramatic reduction in life span in the absence of *lin-42*, it is worth noting that, conversely, overexpression of *lin-42* significantly extended *C. elegans* life span (Fig. 6B and 6E). Notably, the strong correlation between autophagic flux and *PER2* expression during aging in human fibroblasts, along with the newly disclosed connection between *lin-42* and life span extension, indicate a role for *lin-42* in autophagy regulation. In support of a causative role for *lin-42*-regulated autophagy in a “rejuvenating” process, reducing the post-developmental expression of key autophagy genes in *C. elegans*, such as *bec-1*/Beclin and *atg-5*/ATG5, which as expected slightly reduced the life span of wild-type animals, completely suppressed the life span extension effect elicited by *lin-42* overexpression (Fig. 6C-E). Most importantly, we observed that suppression of *lin-42* led to the accumulation of autophagosomal foci in a *C. elegans* transgenic strain expressing the mammalian LC3 orthologue LGG-1 fused to GFP [28] (Fig. 6F). To establish whether the increased accumulation of LGG-1/LC3::GFP foci is the result of an improved or blocked autophagic flux [33, 34], we utilized a transgenic *C. elegans* strain expressing the mammalian p62 orthologue SQST-1 tagged to GFP under the *sqst-1* promoter (Fig. 6G-H). Indeed, similarly to its mammalian counterpart, SQST-1 is efficiently degraded when an intact autophagic flux is induced [35], while it accumulates when autophagic flux is impaired [36]. We found that suppression of *lin-42* led to increased p62 levels in the worms (Fig. 6G-H), demonstrating that *lin-42* is, like the mammalian homologue *PER2*, a positive regulator of the autophagic flux. Our findings reveal for the first time a conserved role for Period gene-dependent regulation of autophagy in the aging process.

## DISCUSSION

Various studies link mammalian aging to a deterioration of autophagy and circadian clock functions with ample consequences for cellular and organismal physiology [2, 9]. Nevertheless, the hypothesis that these processes might be mechanistically connected has never been addressed. Here we studied human skin cells aged in situ, in which circadian oscillator functions and autophagy levels are experimentally accessible. We analysed the mechanisms underlying age-correlated changes by genetic loss and gain of function experiments in mammalian fibroblasts and *C. elegans*. The central findings of our study are that core clock genes regulate autophagy in an evolutionarily conserved and cell-autonomous manner, and that clock-dependent control of autophagy is disrupted in aged mammalian cells and might in turn regulate the aging process.

In primary human fibroblasts a tight correlation between donor age, autophagic flux and core clock gene expression was observed. In accordance with our findings, age-related deregulation of clock genes has been reported before in several instances [9, 27, 37, 38]. Subsequently, *PER2* was demonstrated to play a role in cell-autonomous autophagy regulation. However, it should presently not be excluded that other clock genes also deregulated in aged fibroblasts likewise play a role in autophagy regulation and aging. It has also been reported that the circadian clock controls autophagy levels in mouse liver via the transcription factor CCAAT/enhancer-binding protein beta (*Cebpb)* [39]. In agreement with this finding we observed a correlation between *CEBPB* expression levels, autophagy, and age in human fibroblasts (Fig. S6A). However, knockdown of *Per2 in* NIH 3T3 fibroblasts reduced autophagy without changing *Cebpb* expression levels (Fig. S6B), indicating that in these cells *Cebpb* is not crucial for the circadian regulation of autophagy, while still serving the regulation of autophagy in response to metabolic changes. One report showing that the transcription of *Cebpb* is enhanced after amino acid starvation would support this notion [40]. In liver, autophagy flux is maximal in the middle of the light phase [39] while in skin cells the maximum is reached in the middle of the dark phase (Fig. 4B). In line with this difference, the contribution of the circadian clock to autophagy regulation might vary depending on the tissue or the metabolic conditions. The circadian regulation of autophagy genes in fasting mice is different from mice fed ad libitum, and autophagy gene expression in skeletal muscle seems to be non-rhythmic [39]. Tissue-specific diversity of circadian gene regulation has been recognized for a long time [41]. Accordingly, clock-dependent autophagy regulation is bound to be different in a fibroblast adapting to actinic stress as compared to a liver cell adapting to feeding-fasting cycles [39] or a skeletal muscle cell adapting to exercise and rest [42].

Emerging evidence indicates that the circadian clock regulates major pathways of autophagy and aging processes. SIRT1 is rhythmic in mammalian cells and organs and most likely driven by the circadian clock [43, 44]. Whereas the specific role of PER2 in the regulation of SIRT1 levels is still unclear, SIRT1 controls PER2 protein levels by deacetylating PER2 and thereby promoting its degradation [44]. Recently, also mTOR signalling and the circadian clock have been shown to reciprocally regulate each other's activity [45]. Interestingly, a metabolic circadian clock regulates mTOR activity in addition to the canonical light-entrainable clock [46]. While the present study did not reveal the molecular mechanism that connects clock gene expression to the autophagy machinery in aging fibroblasts, PER2 as part of the molecular circadian oscillator affects the expression of a large number of genes in mammalian cells [47], which opens the possibility that PER2 exerts direct or indirect transcriptional control over a metabolic pathway that connects autophagy and aging. Various small molecules have been identified that modulate circadian clock function, and some of them affect cellular PER2 levels [48]. It should be of high clinical relevance to find applications for such drugs in autophagy-related pathologies of the skin, e.g. melanoma [49], in particular in aged subjects.

Lin-42, the homologue of mammalian period proteins in the worm C. elegans, was identified as a regulator of autophagy and life span. Both PER2 and lin-42 positively regulate autophagy, and the fact that this role appears largely conserved in a nematode not exhibiting a mammalian type of circadian regulation indicates that this function of Period proteins is possibly not stringently linked to core clock functionality. Previously it had been reported that lack of lin-42 expression leads to abnormal developmental changes due to altered expression of heterochronic genes. The reduced life span of lin-42-deficient worms in our experiments is unlikely to be attributed to a developmental deficit since these mutants reached the fertile stage at the same time as wild-type animals. However, we cannot exclude that the loss of lin-42 leads to developmental defects, which only become evident and kill the animals later in life. In this context it is interesting to note that middle-aged *Per2*-deficient mice have reproductive deficits that are comparable with those seen in aged wild-type mice [26], and these deficits might be due to reduced autophagic flux, which is highly induced in wild-type mice and is essential for survival and correct development of the embryo [50].

It should be noted that *Cry1^−/−^;Cry2^−/−^* and *Per1^−/−^;Per2^m/m^* animals have comparable life spans which are reduced compared to wild type mice [51]. In contrast, we found that loss of both Cryptochromes enhances autophagy levels while reduction of *Per2* expression causes a decline in autophagic flux. This discrepancy might be explained by detrimental systemic effects on physiology and metabolism that are dominant over effects on autophagy in total knockout animals of Period or Cryptochrome genes. Moreover, we limited our study to fibroblasts while the knockout mice used by Lee at al (2010) lack *Per* or *Cry* genes in every cell of their body. Our results point directly towards a role of clock genes in skin aging but only imply an influence of clock gene expression on autophagy levels in other tissues and longevity of the whole organism. More extensive studies are therefore required to address these questions directly.

## MATERIALS AND METHODS

### Luminometric analysis of cultured mouse fibroblasts

NIH 3T3 cells stably expressing a luciferase reporter construct of the *Bmal1* promoter (NIH 3T3 Bmal1-Luc) and mouse embryonic fibroblasts (MEFs) isolated from *Cry1^−/−^;Cry2^−/−^* mice or wild type littermates were cultured in Dulbecco's modified Eagle's medium (DMEM) supplemented with 10% fetal bovine serum (FBS) and 1% penicillin/streptomycin (Life Technologies, Darmstadt, Germany). Primary human dermal fibroblasts were transduced with *Bmal1-Luc* expressing lentiviruses according to previously published methods [19]. For continuous circadian monitoring of *Bmal1* promoter activity, cells were synchronised for 30 min with 100 nM dexamethasone and re-seeded in phenol red-free medium supplemented with 100 μM luciferin. Real time bioluminescence of the *Bmal1* reporter gene was monitored in a light tight incubator equipped with photomultiplier tube detector assemblies (LumiCycler, Actimetrics, Wilmette, Illinois, USA). Period length, phase and amplitude of circadian oscillations were evaluated using the computer software supplied with the LumiCycler [52].

### Culture of primary human dermal fibroblasts

Human dermal fibroblasts were isolated from skin specimen acquired in the course of cosmetic surgery from sun-protected areas at the bottom side of human female breast from donors aged 20-67. Isolation and primary culture of primary human dermal fibroblasts followed published procedures [13]. Cells were expanded up to population doubling = 9, at which telomere shortening was not yet significant. Replicative cell cycle arrest was determined to occur at population doubling > 50.

### siRNA-mediated knockdown

For siRNA-mediated knockdown experiments 2 × 10^5^ cells were seeded into 35 mm culture dishes. Cells were transfected with ON-TARGETplus smartpool siRNA (Thermo Scientific, Schwerte, Germany). ON-TARGETplus Non-targeting siRNA (Nt) was used as a control. Transfection was performed according to manufacturer's instructions.

### mRNA analyses

For circadian quantification of mRNA abundance cells were harvested 26, 30, 34, 38, 42 and 46 h after synchronization with dexamethasone. RNA was isolated and reversely transcribed using RNeasy mini extraction and Quantitect Reverse Transcription kits (Qiagen, Hilden, Germany). Quantitative RT-PCR was carried out using the LightCycler480II detection system and LightCycler480 Probes master mix (Roche, Penzberg, Germany). Relative crossing points (ΔCP) are calculated from cycle thresholds (CT) according to ΔCP = 2E-ΔCT and ΔCT = CT(gene of interest) – mean CT(3 housekeeping genes).

### Transfection of primary human dermal fibroblasts

Expression of PER2 in human dermal fibroblasts was performed using the P2 Primary Cell 4D Nucleofector X Kit (Lonza, Switzerland) following manufacturer's instructions. 2×10^6^ cells were suspended in 100 μl nucleofection solution together with 2.5 μg PER2-GFP or GFP vector and transferred into a nucleofection cuvette. Program FF-113 was used for transfection followed by incubation for 10 min at room temperature. 400 μl culture medium was added to the cells which were subsequently transferred into 6-well plates or 8-well Ibidi slides for microscopy.

### Protein detection and autophagy measurements

Proteins were quantified by immunoblotting using rabbit antibodies against LC3B, ULK1 (Cell Signaling Technologies, Danvers, MA, USA), PER2 (Millipore, Darmstadt, Germany) and BMAL1 (Abcam, Cambridge, UK). Gel loading was equalized according to protein content of the samples and controlled by amido black staining of the blotted membranes or parallel immune staining using rabbit and mouse beta-Actin or rabbit Calnexin antibodies (Sigma Aldrich, Martinsried, Germany). Western blots were developed with Peroxidase-coupled secondary antibodies. Chemo-luminescence was quantified with a digital camera system (LAS 4000, Fuji, Düsseldorf, Germany). Autophagy flux was determined according to current guidelines by treating cells with chloroquine (50 μM, 3 h) or bafilomycin (10 nM, 3 h) prior to the analysis of LC3-II protein levels. Autophagic flux values were calculated by subtracting LC3-II levels in untreated cells from LC3-II levels in cells treated with the lysosome inhibitors Chloroquine or Bafilomycin [16].

### Immunostaining

Cells were fixed with 4% formaldehyde for 10 min and then permeabilized with 0.5% Triton X-100 for 15 min. After blocking LC3B antibody in 0.1% blocking solution was added for 1h and then washed off. The secondary antibody Cy3 (goat anti-rabbit) was added for 1h, washed off again, then mounting medium with DAPI was added. Pictures were taken randomly and quantified with Image J. All steps were carried out at room temperature.

### Senescence-associated (SA)-β-galactosidase staining

2 × 10^4^ cells/chamber were seeded in 500 μl culture medium in 4-chamber slides and incubated over night at 37°C, 5% CO_2_ at saturated humidity. Thereafter cells were washed twice with PBS and fixed with 4% paraformaldehyde for 30 min at 37°C, followed by an additional washing step with PBS. X-Gal stock solution (5% in DMF) was diluted 1:4 in X-Gal dilution buffer (5mM Potassium Ferricyanide Crystalline, 5mM Potassium Ferricyanide Trihydrate, 2mM Magnesium Chloride Hexahydrate in PBS, pH = 6). 500 μl X-Gal working solution/chamber or X-Gal dilution buffer was applied to the cells and slides were incubated at 37°C over night. Slides were washed 3 times for 5 minutes in PBS and mounted with Aqua-Poly/Mount (Polyscience, Inc., PA, USA). Pictures were taken using a Zeiss Axiophot (Carl Zeiss Microscopy GmbH, Germany).

### *C. elegans* strains and maintenance

Animals were kept (semi-)synchronized (by egg lay) at 20°C on Nematode Growth Media (NGM) plates and fed with *Escherichia coli* OP50. For the experiments, worms were synchronized on plates with *E. coli* HT115, a strain used for RNA interference (RNAi). Bacteria were grown in LB medium at 37°C overnight. *C. elegans* strains used for the experiments were obtained from the Caenorhabditis Genetics Center: N2: wild-type, ALF62: bafIs62 [*lin-42*p::gfp + *unc-119*(+)], HZ589: bpIs151[(*sqst-1*::gfp; *unc-76*)IV]; *him-5*(e1490)V, DA2123: adIs2122 [*lgg-1*p::gfp::*lgg-1* + rol-6(su1006)].

### Life span assay

Life span analysis started from a synchronized population of eggs. From the third day after the egg lay worms were transferred to fresh NGM plates every day during the fertile phase (from day 3 to day 6) to prevent starvation and contamination by the progeny. After this time animals were transferred every second or third day on fresh plates. While transferring the number of worms alive and dead was counted. Animals that did not move spontaneously nor responded to a manual stimulus or did not show any sign of pharyngeal pumping were scored as dead. Worms showing an egg laying defect (bags) or an exploded vulva, or that died on the wall or were lost during the lifespan, were censored. Animals used for *atg-5* or *bec-1* RNAi life span were grown on empty vector (HT115) until L4 stage, and then transferred on the correspondent RNAi plates for the rest of the life span. The survival curve was calculated from the dead and censored animals using a Kaplan-Meier estimator for lifespan curves and the p values was calculated using the log-rank test between pooled populations of animals.

### Autophagy measurement in *C. elegans*

LGG-1/LC3::GFP puncta from DA2123 transgenic animals were counted in the hypodermal seam cells of L3 animals, at least 4 different cells were scored in 10-12 different worms randomly chosen on the slide. Worms were mounted on 2% agarose pad and anesthetized with Sodium Azide 20mM. The LGG-1/LC3::GFP puncta were counted on a Zeiss Axio Imager 2 microscope using a 630-fold magnification. Three independent biological replica were carried out. p62/SQST-1 GFP puncta from HZ589 transgenic animals were counted in 15–20 different young adult worms mounted on 2% agarose pad and anesthetized with Sodium Azide 30mM. GFP foci were counted in the anterior pharyngeal bulb. The worms were visualized using 1000-fold magnification on a Zeiss Axio Imager 2 microscope. At least four independent biological trials were carried out.

### Statistics

Results of continuous variables are given as mean ± SEM. GraphPad PRISM version 5.0 (GraphPad Software Inc., USA) was used for normal data distribution analysis (Shapiro-Wilk test) and calculation of significances (Mann-Whitney U-test). P values < 0.05 were considered significant.

### Declaration of ethical approval of human studies

All donors of skin specimen have given their consent to the study in writing. The investigation conforms to the principles of the Declaration of Helsinki and was approved by the Ethics Committee of the Medical Faculty of the University of Düsseldorf.

## SUPPLEMENTARY MATERIAL FIGURES


