# Supplementary material for: Crosstalk of clock gene expression and autophagy in aging
Source: Aging (Albany NY). 2016 Aug 28;8(9):1876–90. doi: 10.18632/aging.101018 (PMC5076443; doi:10.18632/aging.101018)
Supplement: Supplementary file 1 [file aging-08-1876-s001.pdf]

SUPPLEMENTARY MATERIAL

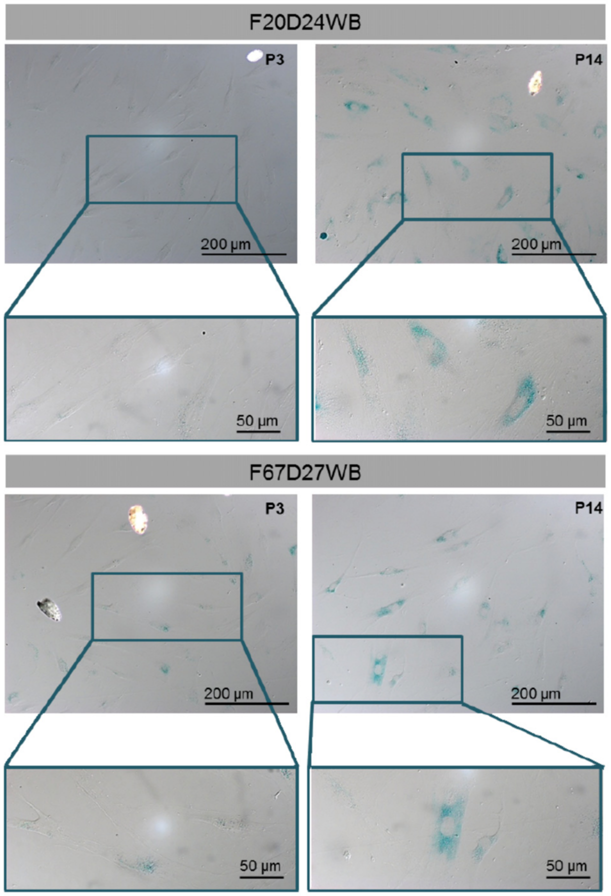

Figure S1. β-galactosidase staining of cells from young and old donors of different passage numbers. Primary human dermal fibroblasts from a young and an old donor were stained for SA-β- galactosidase either at a low passage number (P3) or at a high passage number (P14). Senescent cells stain blue. Pictures were taken using a Zeiss Axiophot (Carl Zeiss Microscopy GmbH, Germany). Size bar 200 or 50 µm.

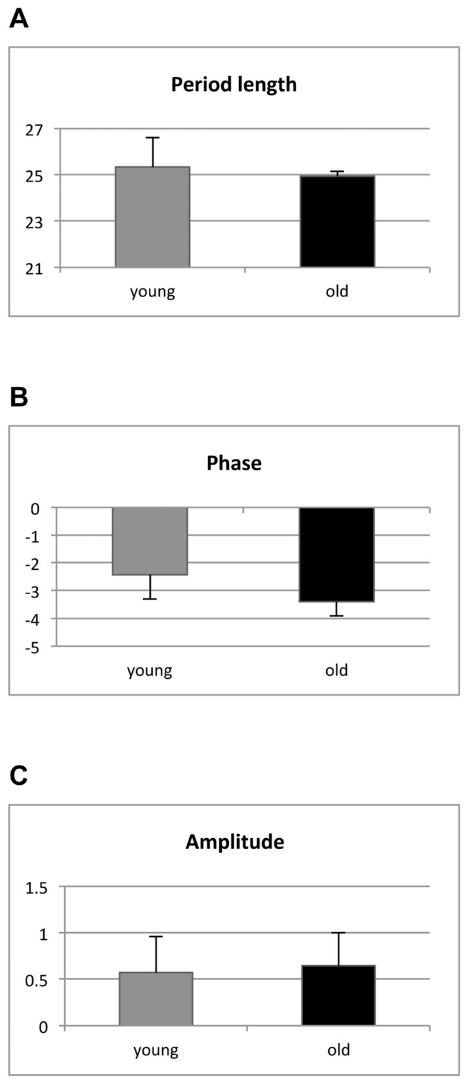

Figure S2. Circadian oscillations in primary human dermal fibroblasts from young and old donors. (A) Period length in hours. (**B**) Phase. (**C**) Amplitude. All data are shown as mean values ± SD, n=3 (young), n=5 (old). All differences between young and old donors are non-significant (unpaired t-test, two-tailed).

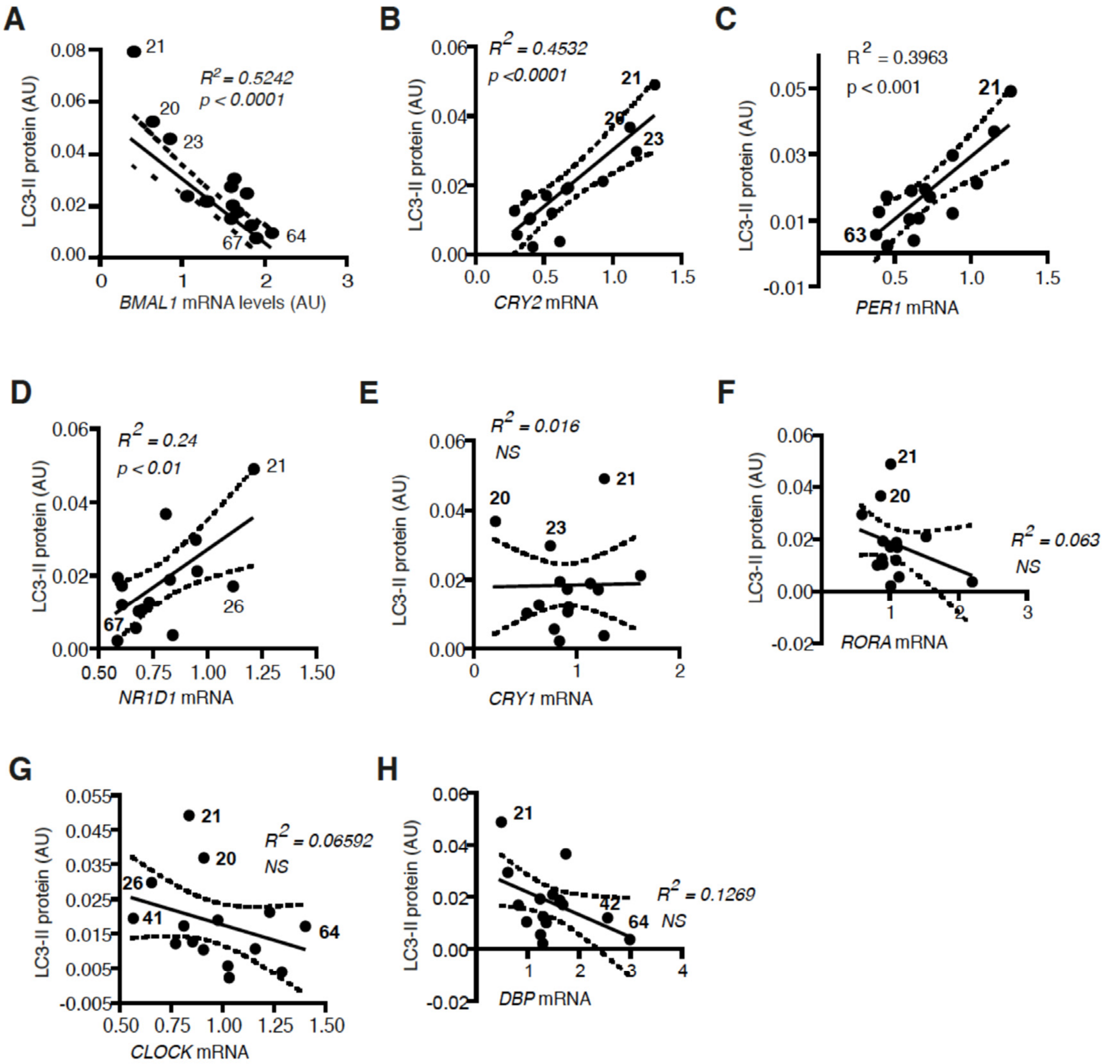

Figure S3. Correlation analyses of core clock gene expression and LC3-II levels. (**A**) BMAL1 (**B**) CRY2 (**C**) PER1 (**D**) NR1D1 (**E**) CRY1 (**F**) RORA (**G**) CLOCK (**H**) DBP.

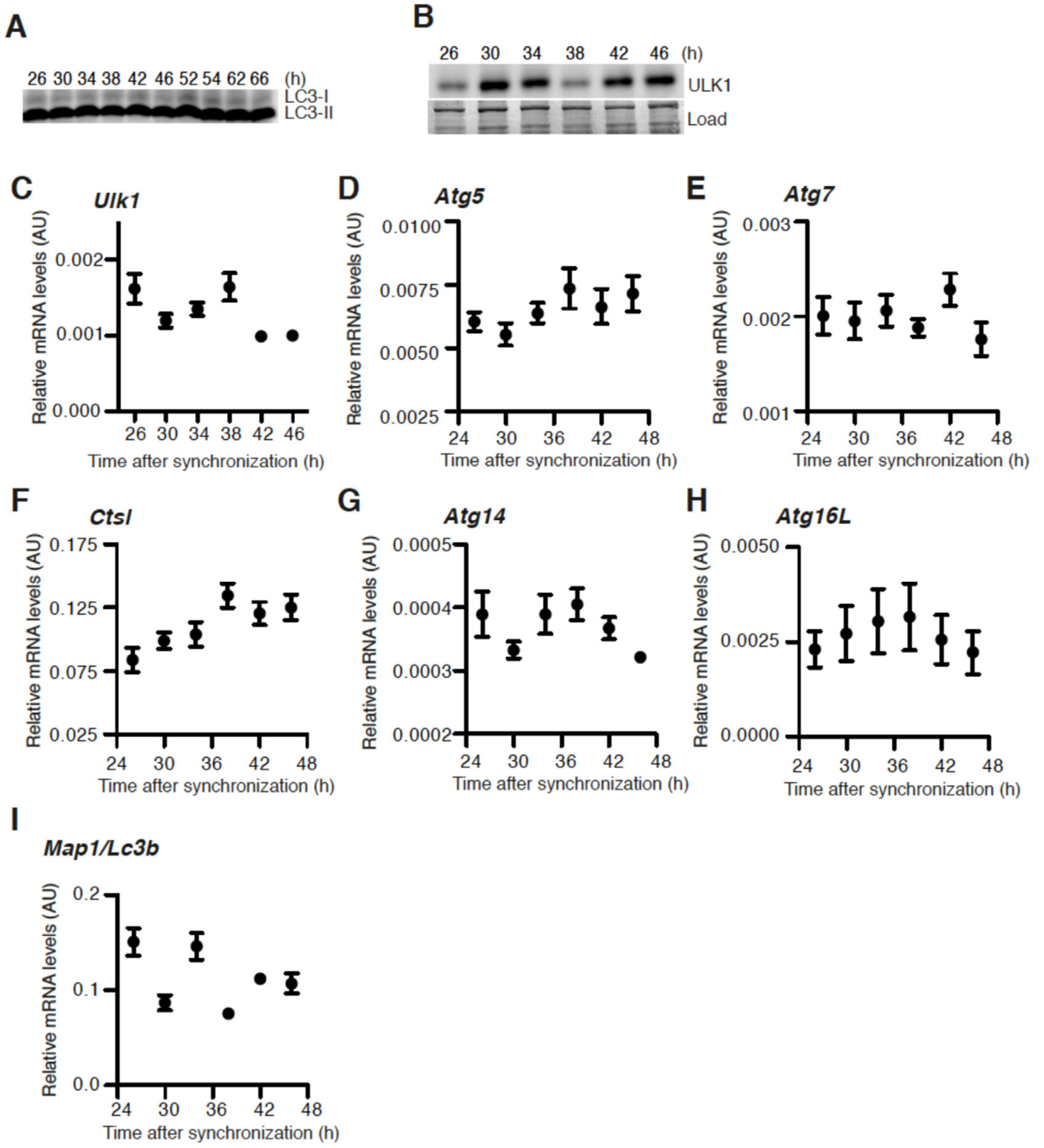

Figure S4. Rhythmic autophagy levels in NIH 3T3 fibroblasts. (**A**) Long exposure of the LC3-II Western blot shown in the upper left panel of Figure 4A. (**B**) ULK1 protein expression. (**C**) *Ulk1* (**D**) *Atg5* (**E**) *Atg7* (**F**) *Ctsl* (**G**) *Atg14* (**H**) *Atg16l* (**I**) *Map1/Lc3b* mRNA expression levels.

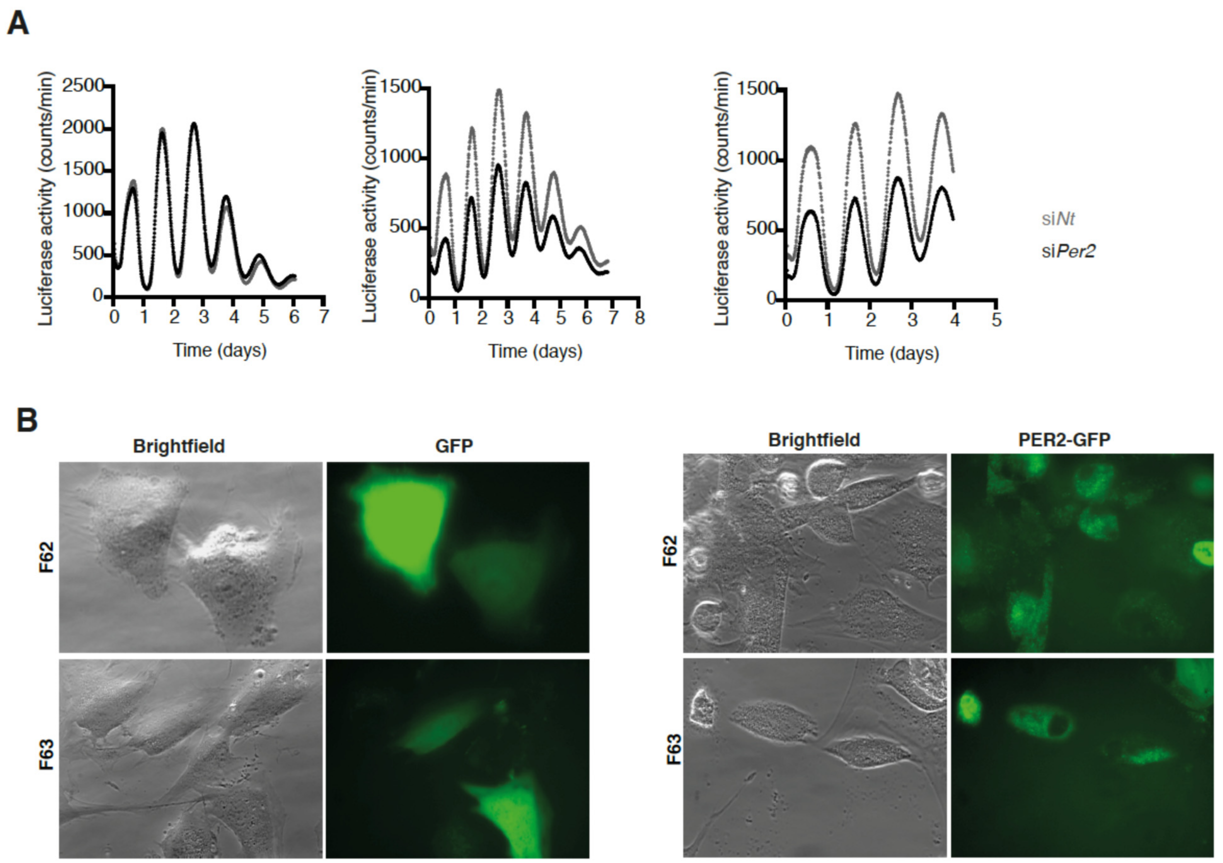

Figure S5. PER2 regulates autophagy. (**A**) In parallel to the knock down experiment shown in Figure 3A *Bmal1-Luc* reporter gene activity was determined in an extra cell culture dish after treatment with siNt (grey) or siPer2 (black). Three biological replicates are shown. (**B**) Fluorescence imaging of primary dermal fibroblasts from aged human donors expressing GFP or PER2-GFP.

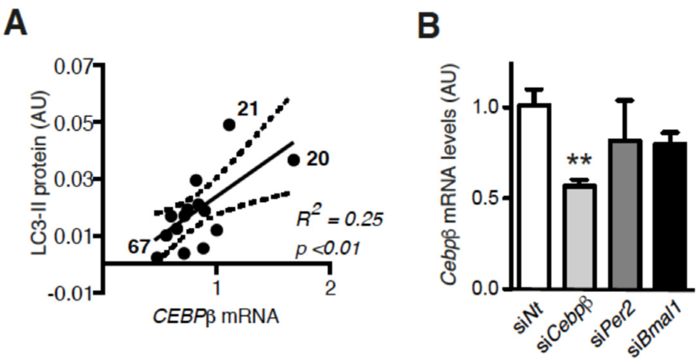

Figure S6. PER2 or BMAL1 do not control Cebpb mRNA levels in NIH3T3 fibroblasts. (**A**) Correlation of *CEBPB* mRNA and LC3-II protein levels in primary human dermal fibroblasts. (**B**) *Cebpb* mRNA levels after siRNA-mediated knock down of *Cebpb*, *Per2* or *Bmal1*. Data are shown as mean values ± SEM, n=4. sterisks designate statistically significant differences of specific siRNA treatment versus control treatment with siNt (unpaired t-test, two-tailed).
